# Responsiveness of the Traumatic Brain Injury Quality of Life Cognition Banks in Recent Brain Injury

**DOI:** 10.3389/fnhum.2022.763311

**Published:** 2022-03-04

**Authors:** Callie E. Tyner, Pamela A. Kisala, Aaron J. Boulton, Mark Sherer, Nancy D. Chiaravalloti, Angelle M. Sander, Tamara Bushnik, David S. Tulsky

**Affiliations:** ^1^Center for Health Assessment Research and Translation, University of Delaware, Newark, DE, United States; ^2^TIRR Memorial Hermann Research Center, Houston, TX, United States; ^3^Baylor College of Medicine, Houston, TX, United States; ^4^Kessler Foundation, East Hanover, NJ, United States; ^5^Department of Physical Medicine and Rehabilitation, Rutgers New Jersey Medical School, Newark, NJ, United States; ^6^NYU School of Medicine, New York, NY, United States; ^7^NYU Langone Medical Center, New York, NY, United States; ^8^Department of Physical Therapy, University of Delaware, Newark, DE, United States; ^9^Department of Psychological and Brain Sciences, University of Delaware, Newark, DE, United States

**Keywords:** acquired brain injury, rehabilitation, assessment, patient-reported outcomes, psychometrics, cognition

## Abstract

Patient report of functioning is one component of the neurocognitive exam following traumatic brain injury, and standardized patient-reported outcomes measures are useful to track outcomes during rehabilitation. The Traumatic Brain Injury Quality of Life measurement system (TBI-QOL) is a TBI-specific extension of the PROMIS and Neuro-QoL measurement systems that includes 20 item banks across physical, emotional, social, and cognitive domains. Previous research has evaluated the responsiveness of the TBI-QOL measures in community-dwelling individuals and found clinically important change over a 6-month assessment interval in a sample of individuals who were on average 5 years post-injury. In the present study, we report on the responsiveness of the TBI-QOL Cognition–General Concerns and Executive Function item bank scores and the Cognitive Health Composite scores in a recently injured sample over a 1-year study period. Data from 128 participants with complicated mild, moderate, or severe TBI within the previous 6 months were evaluated. The majority of the sample was male, white, and non-Hispanic. The participants were 18–92 years of age and were first evaluated from 0 to 5 months post-injury. Eighty participants completed the 1-year follow-up assessment. Results show acceptable standard response mean values (0.47–0.51) for all measures and minimal detectable change values ranging from 8.2 to 8.8 T-score points for Cognition–General Concerns and Executive Functioning measures. Anchor rating analysis revealed that changes in scores on the Executive Function item bank and the Cognitive Health Composite were meaningfully associated with participant-reported changes in the areas of attention, multitasking, and memory. Evaluation of change score differences by a variety of clinical indicators demonstrated a small but significant difference in the three TBI-QOL change scores by TBI injury severity grouping. These results support the responsiveness of the TBI-QOL cognition measures in newly injured individuals and provides information on the minimal important differences for the TBI-QOL cognition measures, which can be used for score interpretation by clinicians and researchers seeking patient-reported outcome measures of self-reported cognitive QOL after TBI.

## Introduction

Cognitive concerns are common after traumatic brain injury (TBI), although the presentation of subjective complaints and objective impairments are heterogeneous across individuals (Rabinowitz and Levin, 2014). Post-injury assessment of objective cognitive functioning requires a thorough test battery, typically including performance-based measures of neuropsychological functioning—particularly in the domains of attention, executive functioning, and memory—, caregiver or collateral ratings of symptoms and functioning, and behavioral observations (Lezak et al., 2012). Subjective patient report of symptoms and functioning are useful to contextualize the objective data and collateral ratings, particularly since both amplification of deficits and lack of awareness of deficits have been observed after TBI (Flashman and McAllister, 2002; Jamora et al., 2012).

Patterns of post-injury recovery of cognitive symptoms have been shown to differ with injury severity, mechanism, and location, with the fastest rate of recovery for both objective cognitive impairments and subjective report of symptoms generally observed in the first 6 months post-injury (Cristofori and Levin, 2015; Model Systems Knowledge Translation Center, 2019). Cognitive symptoms typically resolve rapidly after mild TBI but may be long-lasting—even lifelong—after severe TBI (Schretlen and Shapiro, 2003; Iaccarino et al., 2015).

Subjective experience of cognitive difficulties post-TBI can be evaluated *via* interview, although formal assessments of patient-reported outcomes (PROs) are useful for settings where standardization is needed (Reeve et al., 2013). Standardization allows for repeated assessment of an individual, which is valuable for monitoring progress in rehabilitation or fluctuation of symptoms over time (Snyder et al., 2012). Standardized PROs are also vital for comparing groups as part of controlled treatment trials (Brundage et al., 2013). Condition-specific PROs can be particularly useful in these settings as they provide a shared context for specificity of the item content and also a relevant clinical comparison sample for score interpretation (Schifferdecker et al., 2018).

Several initiatives have been undertaken in the United States over the past two decades to develop standardized PRO measures for use in healthcare settings. This includes most notably the Patient Reported Outcomes Measurement Information System (PROMIS^®^) initiative funded by the National Institutes of Health Common Fund and the Quality of Life in Neurological Disorders (Neuro-QoL™) measurement system funded by the National Institute of Neurologic Disorders and Stroke (Fries et al., 2005; Cella et al., 2006, 2007, 2011; National Institutes of Health, 2015; Northwestern University, 2021a). PROMIS and Neuro-QoL measures and their supporting documentation are freely available on the HealthMeasures.net website (Northwestern University, 2021a).

The Traumatic Brain Injury Quality of Life measurement system (TBI-QOL) (Tulsky et al., 2016a; Tulsky and Kisala, 2019) is a TBI-specific measurement system targeted to domains and issues relevant to individuals with TBI. It is composed of item banks designed specifically for individuals with TBI (e.g., Headache Pain, Independence, and Grief/Loss) as well as generic item banks that are relevant to most individuals (e.g., Depression and Anxiety). For the generic item banks, the TBI-QOL measures are optimized versions of the PROMIS and Neuro-QoL measurement systems. The TBI-QOL was developed as part of TBI Model System Collaborative research project beginning in 2007 with support from the National Institute on Disability, Independent Living, and Rehabilitation Research (formerly the National Institute on Disability and Rehabilitation Research) (Tulsky et al., 2016a). The foundation of the content domains and item content included in the TBI-QOL measures are grounded in qualitative work with TBI patients, clinicians, and caregivers (Carlozzi et al., 2011). The TBI-QOL includes 20 item banks across physical, emotional, social, and cognitive domains (Tulsky and Kisala, 2019). All TBI-QOL item banks have been calibrated using item response theory (IRT) and are available as computer adaptive tests (CATs) that can rapidly evaluate and monitor individuals following TBI (Tulsky et al., 2016b,^2019^; Capo-Lugo et al., 2019; Carlozzi et al., 2019, 2020; Cohen et al., 2019; Kisala et al., 2019, 2020; Heinemann et al., 2020; Sherer et al., 2020). Composite scores for each of the four domains and for global QOL are also available (Tyner et al., 2020). The TBI-QOL measures can be accessed and administered as fixed-length forms or CATs *via* REDCap (Harris et al., 2009, 2019), or through the NIH Toolbox and PROMIS iPad Apps (Northwestern University, 2021b). Furthermore, the item banks and short forms can be obtained by contacting the authors at TBI-QOL@udel.edu. Details on the TBI-QOL measures and composite scores are presented in Table 1.

**TABLE 1:** Traumatic Brain Injury Quality of Life (TBI-QOL) item bank content, item counts, and scoring information.

Item banks	Item content	Number of items in bank	Number of items in short form	Interpretation direction (higher scores)	Range of possible scores for short form^a,b^
Mobility	Ability to carry out bodily movements requiring ambulation, balance, or endurance	32	9	Better function	19.6–59.9
Upper extremity	Ability to carry out various activities involving digital, manual, and reach-related functions	33	9	Better function	15.8–54.9
Fatigue	Feeling of low energy, interference with tasks	73	10	Severe symptoms	32.7–80.7
Pain interference	Degree of pain impact on relationships, work, mood	40	10	Severe symptoms	40.4–78.8
Headache pain	Experience of headache symptoms and impact on daily functions	13	10	Severe symptoms	38.9–72.6
Positive affect and well-being	Sense of well-being, life purpose, meaning, and satisfaction	32	9	Better function	25.4–67.8
Depression	Sadness, guilt, self-criticism, worthlessness, loneliness, alienation, and loss of interest	28	8	Severe symptoms	38.3–82.6
Anxiety	Worry, fear, nervousness, and tension	28	10	Severe symptoms	36.0–85.1
Stigma	Experience of negativity, prejudice, and discrimination post-injury	28	10	Severe symptoms	37.1–79.8
Resilience	Experience and process of successfully adapting to challenging experiences	27	10	Better function	15.3–69.2
Grief/loss	Emotional reactions to grief including anger, guilty, anxiety, sadness, and despair	17	9	Severe symptoms	32.9–75.0
Self-esteem	Emotional, evaluative, and cognitive perceptions of competence and worth	13	10	Better function	20.0–64.9
Anger	Frustration, aggression, irritability, hostility, and interpersonal sensitivity	35	10	Severe symptoms	37.1–86.7
Emotional and behavioral dyscontrol	Disinhibition, emotional lability, irritability, impatience, and impulsiveness	26	10	Severe symptoms	33.1–84.6
Executive function	Difficulties with planning, organizing, calculating, and problem solving	37	10	Better function	12.3–58.0
Cognition–general concerns	Difficulties with learning, memory, attention, and concentration	39	10	Better function	17.3–59.6
Communication/comprehension	Difficulties with language expression, articulation, and comprehension,	31	9	Better function	12.8–67.2
Ability to participate in social roles and activities	Degree of symptom interference in social functioning, including work, family, friends, and leisure	45	10	Better function	25.9–60.7
Satisfaction with social roles and activities	Amount of enjoyment with regard to social functioning, including work, family, friends, and leisure	41	10	Better function	28.2–61.2
Independence	Autonomy, ability to assert oneself, sense of control over one’s life	13	8	Better function	21.2–66.2

**Composite scores**	**Source of scores**			**Interpretation direction (higher scores)**	**Range of possible scores** *^c^*

Physical health	Fatigue; pain interference			Better function	60–140
Emotional health	Anger; anxiety; depression			Better function	57–141
Cognitive health	Cognition–general concerns; executive function			Better function	57–130
Social health	Ability to participate in social roles and activities; satisfaction with social roles and activities			Better function	57–122
Global QOL	Physical health composite; emotional health composite; cognitive health composite; social health composite			Better function	57–145

*All TBI-QOL items, parameters, and data are © 2016 David Tulsky. All rights reserved. All TBI-QOL items originally from Neuro-QoL are © 2008–2013 David Cella on behalf of the National Institute for Neurological Disorders and Stroke. All items are freely available to the public upon request (contact TBI-QOL@udel.edu).*

*^a^Item bank scores are in T-score units (M = 50, SD = 10).*

*^b^While the short forms have a predefined possible score range, the CAT administration may yield scores outside of this range depending on the items administered.*

*^c^Composites are in standard score units (M = 100, SD = 15).*

The TBI-QOL cognition item banks are of particular interest for neuropsychological rehabilitation, given the need for standardized, reliable, and validated outcomes measures that can evaluate subjective symptoms during and following rehabilitation. The TBI-QOL cognition item banks are therefore the focus of this psychometric study. These banks include newly written items as well as items that were drawn from the Neuro-QoL v1.0 cognition item banks. A large effort of qualitative work in TBI was undertaken to develop new TBI-specific items to build these new measures (Carlozzi et al., 2019). With the newly written items, additional focus was placed on the domains of problem solving, cognitive flexibility, learning/memory, attention/concentration, and processing speed (Carlozzi et al., 2019). The structure of the item division into two banks (Executive Function and Cognition–General Concerns) was maintained to preserve cross-condition comparability with the Neuro-QoL v1.0 item banks of the same name. All items were calibrated using IRT based on data gathered from individuals with complicated mild, moderate, or severe TBI (Tulsky et al., 2016a; Tulsky and Kisala, 2019). Thus, both the TBI-QOL Cognition–General Concerns and TBI-QOL Executive Function item banks are optimized for people with TBI. The scores on these two TBI-QOL cognition measures have been transformed and equated to the Neuro-QoL item banks so that both of these item banks are directly comparable to a score on the parallel Neuro-QoL v1.0 item bank. The strong test-retest reliability of the TBI-QOL cognition measures has been established previously [i.e., Executive Function: *r* = 0.86 (95% CI = 0.79–0.91); Cognition–General Concerns: *r* = 0.88 (95% CI = 0.81–0.92)] (Carlozzi et al., 2019).

The responsiveness of a PRO measure is an aspect of construct validity that estimates the ability of a measure to capture meaningful change over time (Liang, 2000; Terwee et al., 2003; Revicki et al., 2006; King, 2011). Minimal important differences (MIDs) can be computed to facilitate interpretation of changes in PRO scores over time (Revicki et al., 2008). Neither responsiveness nor MID are fixed characteristics of PRO measures, as they each will vary in different groups and settings. For a PRO to be recommended for a specific use, the responsiveness of the measure to detect meaningful change must be demonstrated in the target population (Roach, 2006). Thus, use of multiple methods of evaluation is recommended to lend support to responsiveness of a measure (Revicki et al., 2008). Preliminary evidence of responsiveness of the TBI-QOL measures have been shown in a community-dwelling sample comprised of individuals who were an average of 5 years post-injury (Poritz et al., 2020). This study showed general stability of TBI-QOL scores over a 6-month study period, with a small but significant improvement in TBI-QOL Executive Functioning item bank scores (i.e., average of 2.7 T-score points) observed in individuals who reported improved participation across the 6-month study period on the Participation Assessment with Recombined Tools–Objective (PART-O) (Poritz et al., 2020).

As part of the process of marshaling evidence for the construct validity and utility of the TBI-QOL, the present study was designed to evaluate the responsiveness of the TBI-QOL cognition measures in a sample of individuals with recent TBI (i.e., <6 months post-injury). Patient report of cognitive functioning and symptoms from this sample provides a unique opportunity for evaluating measure responsiveness, as improvement in self-reported cognition functioning would be expected for many individuals due to the typical course of recovery after TBI. Additionally, this will be the first study to evaluate the responsiveness of the TBI-QOL Cognitive Health Composite score, which is one of five recently developed summary metrics for the TBI-QOL measures (Tyner et al., 2020). Although there is some disagreement about the best metrics to use for evaluating the responsiveness of outcomes measures (Angst, 2011), this study sought to examine responsiveness following the recommendations of the COSMIN guidelines (Terwee et al., 2003; Mokkink et al., 2010), which recommend testing differences in change scores between groups (Mokkink et al., 2010), in addition to evaluating both distribution- and anchor-based metrics of MID (Revicki et al., 2008; King, 2011).

## Materials and Methods

### Participants

Participants were 134 adults with a history of recent TBI recruited from three clinical sites in the United States as part of a larger multi-year study to evaluate longitudinal trajectories of recovery after TBI. All three sites were part of the TBI Model Systems program, which is a network of institutions that are funded by the United States National Institute on Disability, Independent Living, and Rehabilitation Research as leading centers for clinical care and research on TBI (Model Systems Knowledge Translation Center, 2021). Criteria for enrollment in the TBI Model Systems are described elsewhere and include meeting a standard definition of a moderate or severe TBI with objective medical indicators and being age 16 or older at time of injury (National Data and Statistical Center of the Traumatic Brain Injury Model System, 2007; Corrigan et al., 2012). This study also included individuals with a history of complicated mild TBI, as defined by the presence of abnormal structural neuroimaging in the context of an otherwise mild TBI. Only participants injured less than 6 months prior to enrollment were included in the present study. Only participants with capacity to provide informed consent, as determined by the referring physician or psychologist, were enrolled. Additional inclusion criteria for this study were age 18 or older at time of consent, ability to read, speak and understand English, and ability to understand and respond (by voice) to PRO items. Medical records were reviewed to confirm diagnosis of TBI. Injury severity was categorized as complicated mild, moderate, or severe based on available medical record information (i.e., Glasgow Coma Scale score in emergency department, duration of loss of consciousness, duration of post-traumatic amnesia, and neuroimaging findings) using standard classification schema (Williams et al., 1990; O’Neil et al., 2013). The institutional review board or ethics committee at each site reviewed and approved this project.

### Measures and Procedures

Data collection was conducted either as part of an in-person or telephone interview where participants received select measures from the TBI-QOL (i.e., Depression, Anxiety, Resilience, Anger, Emotional and Behavioral Dyscontrol, Headache Pain, Fatigue, Executive Functioning, and Cognition–General Concerns) as CATs with an eight-item minimum and 12-item maximum. These measures yield T-scores with a mean of 50 and standard deviation (SD) of 10. Since the TBI-QOL Cognition–General Concerns and Executive Function banks have been linked to the Neuro-QoL v1.0 metric, the reference sample for these item banks is a combined general population and clinical neurological sample (Gershon et al., 2012). Additional details on CAT administration, calibration, and normative references for the TBI-QOL measures is provided elsewhere and will not be repeated here (Tulsky and Kisala, 2019).

There are 37 Executive Function and 39 Cognition–General Concerns items calibrated in these banks; the item stems (i.e., the phrasing of each question), item contexts (e.g., “In the past 7 days…”), and response sets (e.g., Never/Rarely/Sometimes/Often/Always) for these two banks have been published previously and will not be repeated here (Carlozzi et al., 2019). The TBI-QOL Cognitive Health Composite score for each assessment was calculated using the lookup tables provided by the developers (Tyner et al., 2020), whereby the T-scores from the Executive Function and Cognition–General Concerns measures are summed and converted to an index score. The composite scores are in standard score units, with a mean of 100 and a SD of 15, and are based on a cohort of persons with TBI (Tyner et al., 2020). Other traditional assessments of resilience, depression, anxiety, participation, life satisfaction, general health, anger, and fatigue were also administered as part of a larger data collection effort and were not included in the present analyses.

Participants completed the TBI-QOL measures in interview format at the initial assessment (i.e., at time of enrollment) and then a second time 1 year later. At the 1-year follow-up assessment, participants also completed a series of anchor items designed to capture participant perception of any change in symptoms or functioning since the initial assessment (Coon and Cook, 2018). This approach for documenting responsiveness of PRO measures is widely used (Revicki et al., 2008; Ousmen et al., 2018), and provides the benefit of centering the patients’ perspective of their functioning. Anchor ratings were made using a 7-point Likert scale coded as: 1 = Much Worse, 2 = Worse, 3 = A Little Worse, 4 = About the Same, 5 = A Little Better, 6 = Better, 7 = Much Better. For the purpose of this study, only the three TBI-QOL cognition scores and 12 anchor items on the topics of health, well-being, and several areas of cognitive functioning and cognition-related symptoms were analyzed. The wide array of anchor items examined in this study were chosen either because they directly reflect the content of the cognition item banks (i.e., self-perception of various cognitive abilities) or because they cover topics known to be associated with cognition (i.e., overall health, emotional well-being, physical health, and fatigue), as has been recommended (Revicki et al., 2008). This broad approach was selected to be able to detect which, if any, symptom areas the TBI-QOL measures are most responsive to.

### Standardization and Quality Control

Data was collected by trained research assistants. Training of all data collectors at the three sites was centralized through a single project investigator (PK) to ensure consistency and standardization across data collectors and sites. Each data collector attended an interactive, web-based training session, passed a certification quiz, and completed multiple practice assessments prior to beginning live data collection. Data collectors met biweekly to review recruitment and accrual progress and discuss any actual or anticipated changes or challenges. Data collectors were trained to not provide assistance to participants in selecting their responses. Instead, interviewers simply read the questions aloud and directed the participant to the appropriate response card to select the response that they felt most closely fit their experience. All data were entered directly by the interviewer into the Assessment Center*^SM^* online platform (Gershon et al., 2010). The use of this platform and the interview format helped to avoid within-interview missing data (i.e., inadvertent skipping of questions by participants). Data were downloaded and reviewed for quality and completeness at least biweekly. We flagged and reviewed any cases that appeared to fall short of quality standards. There was a case of improbable time stamps for data entry that was traced to a single data collector. After extensive investigation and out of an abundance of caution, six baseline and seven 1-year follow-up observations collected by that single data collector were removed from the dataset.

### Data Analyses

Standard error of measurement [SEM; computed as SD × √(1 − *r*)] (Wyrwich et al., 1999; King, 2011) and minimum detectable change (MDC_95%_; computed as 1.96 × √2 × SEM) (Beaton et al., 2001; de Vet et al., 2006; King, 2011) were computed as distribution-based methods for evaluating the MID of the TBI-QOL Cognition–General Concerns, Executive Function, and Cognitive Health Composite scores. The standard response mean (SRM) (Kazis et al., 1989) was computed as a measure of effect size for the mean difference in these three scores from the initial and 1-year follow-up assessments (computed as *M*_*difference*_/SD_*difference*_). SRM values ≥0.3 were considered indicative of a moderate effect size corresponding to meaningful change (Cohen, 1988). Paired sample *t*-tests were used to evaluate the statistical difference between the TBI-QOL scores at the two time points.

Responsiveness was further evaluated by examining associations between each of the 12 anchor item ratings and TBI-QOL change scores (i.e., difference between initial and 1-year follow-up assessment scores on the Cognition–General Concerns, Executive Functioning, and Cognitive Health Composite) using Pearson correlations. Correlation coefficient values ≥0.30 indicated a meaningful, moderate relationship (Coon and Cook, 2018; Ousmen et al., 2018). The Benjamini–Hochberg procedure was used to correct statistical significance values to account for multiple comparisons (Benjamini and Hochberg, 1995).

Associations of the TBI-QOL change scores with clinical indicators of injury severity at the initial assessment were also evaluated as markers of responsiveness. Group differences in change scores by TBI injury severity (i.e., complicated mild, moderate, and severe) were compared using one-way ANOVA. Associations of TBI-QOL change scores with other markers of injury severity and recovery pace (i.e., time since injury; time to follow commands; acute care length of stay (LOS); inpatient rehabilitation LOS; and living situation at time of initial evaluation) were examined using Pearson correlations, independent samples *t*-tests, and exploratory stepwise multiple regression analyses. Exploratory analysis of patterns of variation in TBI-QOL change scores with demographic variables were also evaluated using Pearson correlations (age) and one-way ANOVAs (gender, race, ethnicity, and recruitment site) to understand any potential covariates. All analyses were conducted using SPSS Version 26 (IBM Corp, 2019).

## Results

Data from 128 participants with a medical record confirmed diagnosis of complicated mild (40%), moderate (21%), or severe (31%) TBI with a baseline assessment within 6 months of injury were analyzed. Injury severity was unknown or missing in 8% of cases. The majority of participants in the sample were male (60%), white (69%), and non-Hispanic (73%). The participants ranged in age from 18 to 92 years of age (mean 51.4) and were first evaluated from 0 to 5 months post-injury (mean 2.0 months). Additional demographic and injury severity indicators are shown in Table 2. Eighty participants completed the 1-year follow-up assessment.

**TABLE 2:** Participant demographic and injury characteristics.

	*M*	SD	*N*
Age (years)	51.4	21.3	125
Time since injury (years)	2.0	1.8	128
Time to follow commands (days)	3.9	8.6	90
Acute care length of stay (days)	15.9	12.6	126
Inpatient rehabilitation length of stay (days)	20.3	13.7	123

	**%**		***N***

**TBI injury severity**			
Complicated mild	39.8		51
Moderate	21.1		27
Severe	31.3		40
Unknown/missing	7.8		10
**Gender**			
Female	38.3		49
Male	60.2		77
Missing	1.6		2
**Race**			
White	68.8		88
Black/African American	18.0		23
Asian	4.7		6
Native Hawaiian or other Pacific Islander	0.8		1
Multi-racial	1.6		2
Other	4.7		6
Not provided	1.6		2
**Ethnicity**			
Hispanic	17.2		22
Non-Hispanic	73.4		94
Not provided	9.3		12
**Education**			
8th grade or less	0.8		1
Some high school	7.8		10
Completed high school*	18.0		23
Some college	28.9		37
Bachelor’s degree	15.6		20
Some graduate school	8.6		11
Graduate or professional degree**	20.3		26
**Living situation**			
Initial inpatient rehabilitation	34.4		44
Other rehabilitation or long-term care facility	1.6		2
At a private residence	64.1		82
**Marital status**			
Single, never married	35.2		45
Married	37.5		48
Separated or divorced	17.2		22
Widowed	10.2		13

*Includes GED and vocational HS.

**Includes MS, MA, PhD, MD, DDS, JD, etc.

Mean TBI-QOL cognition measure scores and SEM values for each time point are presented in Table 3. Change scores showed improvements of 4.0 and 3.9 T-score points on average after 1 year on the Cognition–General Concerns and Executive Function measures, respectively. Mean improvement of 7.3 standard score points was observed on the Cognitive Health Composite. MDC_95%_ values were 8.8 and 8.2 T-score points on the Cognition–General Concerns and Executive Function measures. SRM values ranged from 0.47 to 0.51. Paired samples *t*-tests indicated significant improvement in self-reported cognition after 1 year on both of the TBI-QOL cognition item banks and the Cognitive Health Composite (*p* ≤ 0.001).

**TABLE 3:** TBI-QOL cognition scores, distribution-based change metrics, and paired sample *t*-test results.

	Initial assessment (T1)				1-year follow-up (T2)				Difference (T2 − T1)						MDC_95%_
			
	*n*	*M*	SD	SEM	*n*	*M*	SD	SEM	*n*	*M*	SD	SRM	*t*	*p*	
Cognition–General Concerns	125	41.0	9.2	3.2	79	43.7	7.8	2.7	79	4.0	8.4	0.48	4.24	0.001	8.8
Executive Function	125	40.2	7.9	3.0	80	43.1	7.3	2.7	80	3.9	8.2	0.47	4.21	<0.001	8.2
Cognitive Health Composite	125	103.6	15.4	–	79	108.8	13.1	–	79	7.3	14.3	0.51	4.53	<0.001	–

*SEM, standard error of measurement; MDC, minimal detectable change; SRM, standard response mean.*

*SEM = SD × √(1 − r); MDC (95% confidence interval) = 1.96 × √2 × SEM; SRM = M_difference_/SD_difference_; Cognition–General Concerns and Executive Functioning are in T-score units (M = 50, SD = 10) while Cognitive Health Composite is in standard score units (M = 100, SD = 15).*

The majority of participants reported improvement over the preceding year on the 12 anchor items examined. Table 4 shows the mean change in Cognitive Health Composite scores (in standard score units) for individuals who reported Worse, Same, or Better functioning. Pearson correlation results, shown in Table 5, indicate that three of the 12 anchor items were related with change on the TBI-QOL Executive Function measure and the Cognitive Health Composite, but not the Cognition–General Concerns measure. Using the standard of *r* ≥ 0.3 to indicate a meaningful, moderate correlation, three anchor items were found to be associated with meaningful self-reported improvement in cognition after 1 year: ability to pay attention; ability to multitask; and ability to remember. Considering mean change scores for these three anchor items, the observed mean Cognitive Health Composite score changes (as shown in Table 4) ranged from 10.0 to 10.8 standard score points for those reporting improvements in self-reported cognition over the preceding year (i.e., about two-thirds of a SD). For those reporting no change, mean score changes ranged from −1.7 to 2.9, and for those reporting worsening of cognitive function, mean score changes ranged from −20.0 to 4.5 points, although these latter estimates are based on few individuals and are not considered reliable.

**TABLE 4:** TBI-QOL Cognitive Health Composite score mean change by anchor item response groupings.

	Worse			Same			Better		
			
Since the last time you filled out this questionnaire, your…	*n*	*M*	SD	*n*	*M*	SD	*n*	*M*	SD
Overall health is…	3	−2.33	8.39	12	6.00	8.39	64	8.00	14.88
Emotional well-being is…	5	2.60	2.07	18	4.94	12.60	56	8.48	15.38
Physical health is…	6	4.00	9.63	6	6.00	16.40	67	7.72	14.62
Cognitive functioning (or ability to think) is…	4	0.00	11.34	16	5.06	14.07	59	8.41	14.56
Ability to pay attention is…	0	−	−	24	1.21	12.23	55	9.96	14.46
Ability to concentrate is…	0	−	−	24	4.17	13.61	55	8.67	14.53
Ability to multitask is…	1	−20.00	0.00	29	2.93	12.43	49	10.45	14.36
Ability to solve problems is…	0	−	−	23	4.70	12.65	56	8.38	14.94
Ability to learn is…	0	−	−	27	4.44	12.07	52	8.79	15.27
Ability to remember is…	6	4.50	6.44	19	−1.74	10.52	54	10.80	14.76
Ability to communicate with others is…	0	−	−	23	3.65	10.61	56	8.80	15.43
Level of fatigue or tiredness is…	5	−2.80	10.26	31	6.19	12.25	43	9.28	15.71

*Worse groups includes responses “Much Worse,” “Worse,” and “A Little Worse”; better group includes responses “A Little Better,” “Better,” and “Much Better”; Cognitive Health Composite is in standard score units (M = 100, SD = 15).*

**TABLE 5:** Pearson correlations of anchor item responses with TBI-QOL change scores after 1 year.

	Cognition–General Concerns (*n* = 79)	Executive Function (*n* = 80)	Cognitive Health Composite (*n* = 79)
			
Since the last time you filled out this questionnaire, your…	*r*	*r*	*r*
Overall health is…	0.16	0.25	0.20
Emotional well-being is…	0.18	0.17	0.18
Physical health is…	0.01	0.07	0.01
Cognitive functioning (or ability to think) is…	0.22	0.26	0.25
Ability to pay attention is…	0.27	0.34*	0.31*
Ability to concentrate is…	0.22	0.27	0.24
Ability to multitask is…	0.25	0.38*	0.33*
Ability to solve problems is…	0.15	0.23	0.19
Ability to learn is…	0.16	0.23	0.19
Ability to remember is…	0.26	0.33*	0.30*
Ability to communicate with others is…	0.14	0.23	0.18
Level of fatigue or tiredness is…	0.29*	0.28*	0.29*

Anchor item response choices: 1 = Much Worse, 2 = Worse, 3 = A Little Worse, 4 = About the Same, 5 = A Little Better, 6 = Better, 7 = Much Better. *Correlation is significant at the 0.05 level using Benjamini–Hochberg procedure to correct for multiple comparisons; Bolded values are considered meaningful (i.e., ≥0.3).

**FIGURE 1:**
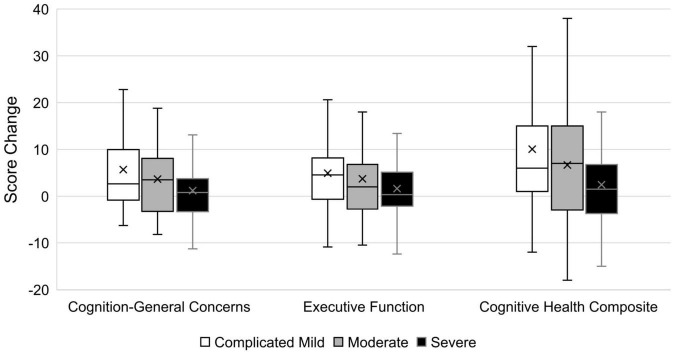
Box and whisker plot of score changes by TBI severity group. Cognition-General Concerns and Executive Functioning are in T-score units (M = 50, SD = 10), while Cognitive Health Composite is in standard score units (M = 100, SD = 15). Box and whisker plot displays error bars to indicate the score range (min to max), with the rectangular box showing the interquartile range, the central line indicating the median value, and the X marking the mean value.

One-way ANOVAs of TBI-QOL change scores by injury severity classification revealed significant (*p* ≤ 0.05) differences in mean change scores by TBI severity group for the Cognition–General Concerns and Cognitive Health Composite (see Table 6), although the effect sizes were small (i.e., 0.23 for both). A box and whisker plot comparing the severity groups is presented in Figure 1. Considering mean change score by injury severity, the complicated mild injury group improved by 0.5–0.7 of a SD over the 1-year study period, the moderate injury group improved by 0.4 of a SD, and the severe injury group improved by 0.1–0.2 of a SD. Tukey *post hoc* contrasts revealed significant differences between the complicated mild and severe groups for Cognition–General Concerns (*p* = 0.025) and the Cognitive Health Composite (*p* = 0.034). To more fully parse the variation in change score by injury severity, the mean score change on the Cognitive Health Composite was compared for individuals of different injury severities by response to the three salient anchor items: attention, multitasking, and memory. Supplementary Table 1 shows that the magnitude of the composite score change (in standard score units) was the largest for those reporting better functioning in the complicated mild group (12.6–15.8), over the moderate (10.7–11.7) and severe (3.8–5.3) groups. Likewise, the mean change score for those reporting same/worse functioning was most negative for the severe group (−2.8 to −1.5) over the moderate (−1.8 to −0.8) group; those in the complicated mild group endorsing same or worse functioning actually showed small improvements on average (1.0–3.6).

**TABLE 6:** TBI-QOL cognition change scores by injury severity.

	Complicated mild			Moderate			Severe			ANOVA		
				
	*n*	*M*	SD	*n*	*M*	SD	*n*	*M*	SD	*F*	ES	*p*
Cognition–General Concerns	36	5.68	9.29	15	3.67	8.24	24	1.20	6.98	4.09	0.23	0.05
Executive Function	36	4.92	8.19	16	3.73	9.66	24	1.62	7.43	2.25	0.17	0.14
Cognitive Health Composite	36	10.06	15.89	15	6.67	13.71	24	2.46	12.06	4.04	0.23	0.05

*For ANOVA, F statistic reported as weighted least squares of linear contrast; ES, effect size; Tukey post hoc contrast revealed significant differences between the complicated mild and severe groups for Cognition–General Concerns (p = 0.025) and the Cognitive Health Composite (p = 0.034); Cognition–General Concerns and Executive Functioning are in T-score units (M = 50, SD = 10) while Cognitive Health Composite is in standard score units (M = 100, SD = 15).*

Evaluation of other markers of injury severity and recovery revealed weak to negligible associations of these clinical variables with TBI-QOL change scores (see Table 7). There were no significant differences in mean change score by different living situations at the initial evaluation [with participants grouped as either (1) living in initial inpatient rehabilitation, other rehabilitation, or long-term care vs. (2) private residence]. To evaluate for potential complex effects of these clinical indicators on patterns of change, exploratory stepwise multiple regression analyses were conducted for the three TBI-QOL change scores, although no models were statistically significant. There were no significant associations or patterns of mean difference observed with any other demographic variables (i.e., correlations between change scores with age and one-way ANOVAs of gender, race, ethnicity, and recruitment site all *p*s > 0.2).

**TABLE 7:** Associations of TBI-QOL cognition change scores with clinical indicators at initial assessment.

	Time since TBI (months; *n* = 79)	Time to follow commands (days; *n* = 57)	Acute care LOS (days; *n* = 79)	Inpatient rehabilitation LOS (days; *n* = 77)	Living situation (*n* = 79)	
					
	*r*	*r*	*r*	*r*	*t*	*p*
Cognition–General Concerns	0.03	−0.11	−0.20	−0.18	0.60	0.55
Executive Function	0.03	0.04	−0.01	−0.14	1.06	0.29
Cognitive Health Composite	0.01	−0.06	−0.13	−0.18	0.91	0.37

*LOS, length of stay. Living situation categorized as two groups [(1) initial inpatient rehabilitation, other rehabilitation, or long-term care vs. (2) private residence]; correlations are non-significant.*

## Discussion

This study provides evidence to support the responsiveness of the TBI-QOL cognition item banks and Cognitive Health Composite score for use in individuals within the first 18 months of recovery post-TBI. Improvements in self-reported cognition were endorsed for the majority of participants in this sample, on both the TBI-QOL cognition measures and using a set of anchor items. Meaningful associations with TBI-QOL Executive Functioning and Cognitive Health Composite score change were observed with anchor ratings of attention, multitasking, and memory. Individuals who endorsed improvements in these domains on anchor ratings improved by about two-thirds of a SD on the TBI-QOL Cognitive Health Composite score. These findings are generally consistent with previous research showing marked improvements in the domains of executive functioning and memory, particularly in the early recovery period, following TBI (Christensen et al., 2008). There were no meaningful correlations between the Cognition–General Concerns change scores and the anchor items, which is unexpected given that the items on this measure were designed to be sensitive to a wide variety of self-reported cognitive symptoms. Although still below the threshold of ≥0.30 to indicate meaningful, moderate relationships, the correlations were statistically significant for all three TBI-QOL measures with fatigue. TBI-QOL score improvements were not consistently associated with self-reported emotional well-being or physical health. The weak association between the TBI-QOL change scores with the anchor ratings of general (non-specific) cognitive functioning, concentration, problem solving, learning, and communication may be due to restriction of range of responses to the anchors. For many of the cognition-related anchor items, no participants endorsed worse functioning. Worse functioning was only reported by four participants for cognitive functioning, one for multitasking, and six for memory. With a larger sample we anticipate that the associations between anchor items and change scores would be more robust.

The MDC values reported in this study are somewhat conservative, as they reflect the amount of change in score where there is 95% certainty statistically that someone would have experienced change in their subjective cognitive functioning. Some authors have suggested that using this 95% certainty criterion is overly conservative in clinical samples, as some individuals who have experienced meaningful change will not be detected (Calfee and Ryan, 2019; Cohen et al., 2021). Using a less conservative method of computation, for example the MDC 90% or using a one-tailed approach, may provide a more accurate estimate of the true MDC (Cohen et al., 2021). For example, in this study the MDC_95%_ values reported (see Table 3) were only met by 15–16% of the sample. If a standard of MDC_90%_ were applied, 7.4 and 6.9 for Cognition–General Concerns and Executive Function, respectively, then 20% of the sample would exceed these thresholds of statistically significant change in self-reported cognitive QOL. These low numbers could be an underestimate of the true number of cases who experienced improved cognitive QOL over this study period. Thus, the MDC values reported in this study should be interpreted with caution in individual cases, given that they are likely overly conservative and some individuals who have experienced important change in cognitive QOL will be missed.

There were small but significant differences in TBI-QOL score changes by injury severity for the Cognition–General Concerns and Cognitive Health Composite, with the greatest score improvement observed in the complicated mild injury group, followed by moderate, and with those in the severe injury group showing the smallest average change scores. This pattern is consistent with previous research showing a greater likelihood for those with mild TBI to return to at or near their pre-injury cognitive status within 6 months to a year, compared to those with moderate or severe injuries where persistent cognitive symptoms are much more common, and recovery takes place over years (Schretlen and Shapiro, 2003; Iaccarino et al., 2015). Furthermore, this finding suggests that without any formal cognitive intervention provided, 1 year may not be long enough to observe changes in self-reported cognitive functioning for newly injured individuals with severe TBI, whereas individuals with complicated mild and moderate injuries are more likely to endorse improvements in cognitive functioning in the first year post-injury in the absence of intervention. This is consistent with previous research showing differences in recovery time course that vary with injury severity, with most individuals after mild TBI showing the bulk of their cognitive symptom improvements in the first few months post-injury and individuals with severe TBI often showing signs of continued improvement as far out as 2 years post-injury (Schretlen and Shapiro, 2003; Iaccarino et al., 2015; Wilkins et al., 2019).

### Limitations

One limitation of this study is that, as an observational study, the expectation for measurable improvement in self-report of cognitive symptoms during the study period may not be as certain as might have been observed had this been a treatment trial. This is demonstrated by the large, and likely overly conservative, MDC values reported. Future research in the context of an intervention study could further support the responsiveness of these measures. Low sample sizes observed in the anchor rating categories, particularly in the worse categories, is a related potential limitation. Given that some amount of cognitive recovery is expected for the majority of individuals following TBI (Schretlen and Shapiro, 2003), we would not have expected many participants to have reported worsening of symptoms at follow-up. Nevertheless, it has been suggested that the presence of low sample sizes in certain anchor categories may be associated with reduced reliability of MID estimates (Ousmen et al., 2018). This study is also limited in that self-report was used for both assessment of changes in cognitive functioning over time and cognitive-related QOL ratings. It is not surprising that people who rate their cognition as worse or unimproved would also rate their cognition-related QOL as poor, and vice versa. Future research should determine the association between objective cognitive test performance and cognitive-related QOL. Furthermore, impairments in self-awareness are common during the early recovery period, particularly in persons with more severe injuries (Flashman and McAllister, 2002). Impaired awareness should be considered in future research investigating subjective reports of cognitive functioning and QOL. Moreover, the high rate of attrition observed at the 1-year follow-up further limits the current results. Therefore, replication of these findings, particularly in a sample as part of a controlled treatment trial, is warranted.

## Conclusion

The findings support the responsiveness of the TBI-QOL Cognition–General Concerns, Executive Function, and Cognitive Health Composite score for use in newly injured individuals. The results presented here provide a variety of distribution- and anchor-based metrics of MID that can be used for score interpretation by clinicians and researchers seeking PRO measures of self-reported cognition after TBI.

## Data Availability Statement

The datasets presented in this article are not readily available because a data use agreement must be signed prior to release. Requests to access the datasets should be directed to DT, (dtulsky@udel.edu).

## Ethics Statement

The studies involving human participants were reviewed and approved by the Institutional Review Boards at NYU Langone Medical Center, Kessler Foundation, Baylor College of Medicine, and the University of Delaware. The patients/participants provided their written informed consent to participate in this study.

## Author Contributions

PK, MS, NC, AS, TB, and DT participated in data collection. AB and PK managed the dataset. CT performed the statistical analysis and wrote the first draft of the manuscript. DT, PK, AB, and AS wrote sections of the manuscript. All authors contributed to manuscript revision, read, and approved the submitted version.

## Conflict of Interest

All TBI-QOL items, parameters, and data are ©2016 David Tulsky. All rights reserved. All TBI-QOL items originally from Neuro-QoL are ©2008–2013 David Cella on behalf of the National Institute for Neurological Disorders and Stroke. All items are freely available to the public upon request (contact TBI-QOL@udel.edu). The contents represent original work and have not been published elsewhere.

## Publisher’s Note

All claims expressed in this article are solely those of the authors and do not necessarily represent those of their affiliated organizations, or those of the publisher, the editors and the reviewers. Any product that may be evaluated in this article, or claim that may be made by its manufacturer, is not guaranteed or endorsed by the publisher.

## Abbreviations

CAT: computer adaptive test
ES: effect size
IRT: item response theory
LOS: length of stay
MDC: minimum detectable change
MID: minimal important difference
Neuro-QoL™: Quality of Life in Neurological Disorders
PART-O: Participation Assessment with Recombined Tools–Objective
PRO: patient-reported outcome
PROMIS^®^: Patient Reported Outcomes Measurement Information System
QOL: quality of life
SEM: standard error of measurement
SRM: standard response mean
TBI: traumatic brain injury.
