# Supplementary material for: Responsiveness of the Traumatic Brain Injury Quality of Life Cognition Banks in Recent Brain Injury
Source: Front Hum Neurosci. 2022 Mar 4;16:763311. doi: 10.3389/fnhum.2022.763311 (PMC8931768; doi:10.3389/fnhum.2022.763311)
Supplement: Supplementary file 1 [file Table_1.DOCX]

| Supplemental Table 1. TBI-QOL Cognitive Health Composite Score Mean Change by Injury Severity and Anchor Item Response Grouping | | | | | | | | | | | | | | | | | | | |
| --- | --- | --- | --- | --- | --- | --- | --- | --- | --- | --- | --- | --- | --- | --- | --- | --- | --- | --- | --- |
|  | Complicated Mild | | | |  | Moderate | | | |  | Severe | | | |  | All Severities | | | |
|  | Same/Worse | | Better | |  | Same/Worse | | Better | |  | Same/Worse | | Better | |  | Same/Worse | | Better | |
| Since the last time you filled out this  questionnaire, your… | *M* | *n* | *M* | *n* |  | *M* | *n* | *M* | *n* |  | *M* | *n* | *M* | *n* |  | *M* | *n* | *M* | *n* |
| Ability to pay attention is... | 3.57 | 14 | 14.18 | 22 |  | -1.40 | 5 | 10.70 | 10 |  | -2.80 | 5 | 3.84 | 19 |  | 1.21 | 24 | 9.65 | 51 |
| Ability to multitask is... | 6.07 | 14 | 12.59 | 22 |  | -0.83 | 6 | 11.67 | 9 |  | -1.50 | 10 | 5.29 | 14 |  | 2.17 | 30 | 10.13 | 45 |
| Ability to remember is... | 1.00 | 14 | 15.82 | 22 |  | -1.80 | 5 | 10.90 | 10 |  | -1.83 | 6 | 3.89 | 18 |  | -0.24 | 25 | 10.54 | 50 |
| *Note:* Same/Worse groups includes responses "Much Worse," "Worse," "A Little Worse," and "Same"; Better group includes responses "A Little Better," "Better," and "Much Better"; Cognitive Health Composite is in standard score units (M = 100, SD = 15) | | | | | | | | | | | | | | | | | | | |
